# Lysol

**Published:** 1892-02

**Authors:** 


### Lysol.

Lysol, the new disinfectant and antiseptic, is recommended as promptly arresting the development of micro-organisms. Cramor, Wehmer, Michelsen, and others have successfully employed it in surgery and gynæcology, and Hanel says it is an unusually agreeable agent for the operator. Unna has used it as a plaster mull in various skin affections, and Phillips has tried it with some success in lupus. It has also been advised in rhino-pharyngeal and laryngeal disease, as well as in diseases of the middle and external ear. It is obtained by dissolving the fraction of tar oil, which boils between 190° and 200° C., in fat, and subsequently saponifying with alcohol. It is a clear, brown, oily liquid, and contains 50 per cent. of cresols. It can be mixed readily with water, and forms clear solutions with glycerine, alcohol, chloroform, and various other fluids. Furbinger recommends $\frac{1}{2}$ to 1 per cent. solution for the hands, and $\frac{1}{4}$ per cent. for instruments. It is only $\frac{1}{8}$ as poisonous as carbolic acid, and cheaper. Pee recommends a 1 per cent. solution in midwifery and gynæcology, and says that a 1 to 200 solution destroys streptococci in fifteen minutes. His experience with it has been very favorable.—*N. Y. Med. Record.*

